# 2-(2-Amino-5-methylthia­zol-4-yl)phenol

**DOI:** 10.1107/S160053680903164X

**Published:** 2009-08-15

**Authors:** Li-Min He, Gao Cao, Ai-Xi Hu

**Affiliations:** a College of Pharmacy, Guangdong Pharmaceutical University, Guangzhou 510006, People’s Republic of China; b College of Chemistry and Chemical Engineering, Hunan University, Changsha 410082, People’s Republic of China

## Abstract

In the title compound, C_10_H_10_N_2_OS, the benzene ring is nearly co-planar with the thia­zole ring, making a dihedral angle of 2.1 (2)°. The crystal structure is stabilized by inter­molecular N—H⋯O hydrogen bonds. An intra­molecular O—H⋯N hydrogen bond is also present.

## Related literature

For background to 2-amino-4-aryl­thia­zoles and their wide-ranging anti­fungal activity, see: Hu *et al.* (2008 ▶); Kazzouli *et al.* (2002 ▶); Holla *et al.* (2003 ▶). For a related structure, see: He *et al.* (2006 ▶).
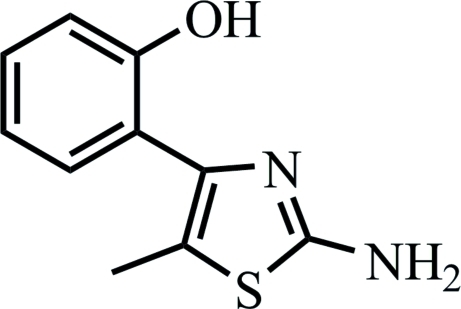


## Experimental

### 

#### Crystal data

C_10_H_10_N_2_OS
*M* *_r_* = 206.27
Orthorhombic, 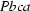

*a* = 12.9391 (5) Å
*b* = 10.3967 (4) Å
*c* = 14.2938 (6) Å
*V* = 1922.86 (13) Å^3^
*Z* = 8
Mo *K*α radiation
μ = 0.30 mm^−1^
*T* = 173 K
0.48 × 0.42 × 0.39 mm

#### Data collection

Bruker SMART 1000 CCD diffractometer
Absorption correction: multi-scan (*SADABS*; Sheldrick, 2004 ▶) *T*_min_ = 0.869, *T*_max_ = 0.891
11037 measured reflections
1881 independent reflections
1706 reflections with *I* > 2σ(*I*)
*R*_int_ = 0.027

#### Refinement

*R*[*F* ^2^ > 2σ(*F* ^2^)] = 0.054
*wR*(*F* ^2^) = 0.150
*S* = 0.98
1881 reflections
129 parameters
H-atom parameters constrained
Δρ_max_ = 1.20 e Å^−3^
Δρ_min_ = −0.33 e Å^−3^

### 

Data collection: *SMART* (Bruker, 2001 ▶); cell refinement: *SAINT-Plus* (Bruker, 2003 ▶); data reduction: *SAINT-Plus*; program(s) used to solve structure: *SHELXTL* (Sheldrick, 2008 ▶); program(s) used to refine structure: *SHELXTL*; molecular graphics: *SHELXTL*; software used to prepare material for publication: *SHELXTL*.

## Supplementary Material

Crystal structure: contains datablocks global, I. DOI: 10.1107/S160053680903164X/xu2563sup1.cif

Structure factors: contains datablocks I. DOI: 10.1107/S160053680903164X/xu2563Isup2.hkl

Additional supplementary materials:  crystallographic information; 3D view; checkCIF report

## Figures and Tables

**Table 1:** Hydrogen-bond geometry (Å, °)

*D*—H⋯*A*	*D*—H	H⋯*A*	*D*⋯*A*	*D*—H⋯*A*
O1—H1⋯N1	0.84	1.77	2.521 (3)	148
N2—H2*B*⋯O1^i^	0.88	2.25	2.961 (3)	138

Symmetry code: (i) 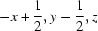.

**Fig. 1.**
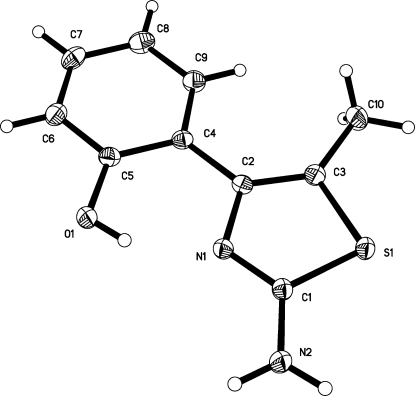
The molecular structure of (I), showing the atom-labelling scheme and 50% probability displacement ellipsoid (arbitrary spheres for H atoms).

## supplementary crystallographic information

### Comment

Compounds containing thiazole are found to exhibit a wide spectrum of biological activities and many of them are well known antiviral, antifungal agents and some are used as pesticides (Kazzouli *et al.*, 2002; Holla *et al.*, 2003; Hu *et al.*, 2008). The structure of 2-amino-4-arylthiazoles was reported before (He *et al.*, 2006). Herein we report the synthesis and crystal structure of the title compound.

The molecular structure of (I) is illustrated in Fig. 1. The molecules are linked by intermolecular hydrogen bonds (N–H···O) and intramolecular hydrogen bonds (O–H···N) (Table 1). The dihedral angle between the planes of thiazole and the benzene ring is 2.1 (2)°.

### Experimental

A solution with 0.005 mol of thiourea and 0.005 mol of 2-bromo-1-(2-hydroxyphenyl)-1-propanone in 50 ml of ethanol was refluxed for 10 h. After finishing the reaction, added 10 ml ammonia and continues to stir the solution 2 h. Then the solution was cooled and the precipitate formed was filtered out, dried, giving white crystals of title compound, yield 60.3%. m.p. 388–389 K. The crystals for X-ray structure determination were obtained by slow evaporation of an ethanol solution at room temperature.

### Refinement

The hydroxy H atom was positioned geometrically (O–H = 0.84 Å) and refined as riding [*U*_iso_(H) = 1.5 *U*_eq_(O)]. Methyl H atoms were positioned geometrically (C–H = 0.98 Å) and torsion angles refined to fit the electron density [*U*_iso_(H) = 1.5 *U*_eq_(C)]. Other H atoms were placed in calculated positions (N–H 0.88 Å and aromatic C–H = 0.95 Å) and refined as riding [*U*_iso_(H) = 1.2 *U*_eq_(C, N)]. The highest peak in the final difference Fourier map is 0.79 Å apart from H8 atom.

### Crystal data

**Table:**

C_10_H_10_N_2_OS	*F*(000) = 864
*M**_r_* = 206.27	*D*_x_ = 1.425 Mg m^−^^3^
Orthorhombic, *P**b**c**a*	Mo *K*α radiation, λ = 0.71073 Å
Hall symbol: -P 2ac 2ab	Cell parameters from 7684 reflections
*a* = 12.9391 (5) Å	θ = 2.4–27.0°
*b* = 10.3967 (4) Å	µ = 0.30 mm^−^^1^
*c* = 14.2938 (6) Å	*T* = 173 K
*V* = 1922.86 (13) Å^3^	Block, yellow
*Z* = 8	0.48 × 0.42 × 0.39 mm

### Data collection

**Table:**

Bruker SMART 1000 CCD diffractometer	1881 independent reflections
Radiation source: fine-focus sealed tube	1706 reflections with *I* > 2σ(*I*)
graphite	*R*_int_ = 0.027
ω scans	θ_max_ = 26.0°, θ_min_ = 2.9°
Absorption correction: multi-scan (*SADABS*; Sheldrick, 2004)	*h* = −15→14
*T*_min_ = 0.869, *T*_max_ = 0.891	*k* = −12→12
11037 measured reflections	*l* = −17→17

### Refinement

**Table:**

Refinement on *F*^2^	Primary atom site location: structure-invariant direct methods
Least-squares matrix: full	Secondary atom site location: difference Fourier map
*R*[*F*^2^ > 2σ(*F*^2^)] = 0.054	Hydrogen site location: inferred from neighbouring sites
*wR*(*F*^2^) = 0.150	H-atom parameters constrained
*S* = 0.98	*w* = 1/[σ^2^(*F*_o_^2^) + (0.0885*P*)^2^ + 3.3976*P*] where *P* = (*F*_o_^2^ + 2*F*_c_^2^)/3
1881 reflections	(Δ/σ)_max_ < 0.001
129 parameters	Δρ_max_ = 1.20 e Å^−^^3^
0 restraints	Δρ_min_ = −0.33 e Å^−^^3^

### Special details

**Table:**

Experimental. ^1^H NMR (CDCl_3_, 400 MHz): 2.48 (s, 3H, CH_3_), 4.97 (br, 2H, NH_2_), 6.86–7.42(m, 4H, phenyl-H).
Geometry. All e.s.d.'s (except the e.s.d. in the dihedral angle between two l.s. planes) are estimated using the full covariance matrix. The cell e.s.d.'s are taken into account individually in the estimation of e.s.d.'s in distances, angles and torsion angles; correlations between e.s.d.'s in cell parameters are only used when they are defined by crystal symmetry. An approximate (isotropic) treatment of cell e.s.d.'s is used for estimating e.s.d.'s involving l.s. planes.
Refinement. Refinement of *F*^2^ against ALL reflections. The weighted *R*-factor *wR* and goodness of fit *S* are based on *F*^2^, conventional *R*-factors *R* are based on *F*, with *F* set to zero for negative *F*^2^. The threshold expression of *F*^2^ > σ(*F*^2^) is used only for calculating *R*-factors(gt) *etc*. and is not relevant to the choice of reflections for refinement. *R*-factors based on *F*^2^ are statistically about twice as large as those based on *F*, and *R*- factors based on ALL data will be even larger.

### Fractional atomic coordinates and isotropic or equivalent isotropic displacement parameters (Å^2^)

**Table:**

	*x*	*y*	*z*	*U*_iso_*/*U*_eq_	
S1	0.29069 (5)	0.23322 (6)	0.58881 (4)	0.0269 (2)	
C1	0.2598 (2)	0.3782 (2)	0.64120 (16)	0.0242 (5)	
C2	0.43091 (18)	0.4000 (2)	0.61774 (16)	0.0232 (5)	
C3	0.4199 (2)	0.2792 (2)	0.58181 (17)	0.0266 (6)	
C4	0.52468 (18)	0.4802 (2)	0.62670 (16)	0.0241 (5)	
C5	0.52103 (19)	0.6026 (2)	0.66966 (17)	0.0263 (5)	
C6	0.6103 (2)	0.6770 (3)	0.67875 (18)	0.0315 (6)	
H6	0.6066	0.7589	0.7081	0.038*	
C7	0.7037 (2)	0.6329 (3)	0.64567 (19)	0.0344 (6)	
H7	0.7642	0.6840	0.6526	0.041*	
C8	0.7095 (2)	0.5136 (3)	0.6021 (2)	0.0369 (7)	
H8	0.7738	0.4829	0.5790	0.044*	
C9	0.6212 (2)	0.4398 (3)	0.59266 (18)	0.0314 (6)	
H9	0.6261	0.3590	0.5620	0.038*	
C10	0.4945 (2)	0.1822 (3)	0.5439 (2)	0.0413 (7)	
H10A	0.5252	0.2149	0.4859	0.062*	
H10B	0.4579	0.1016	0.5309	0.062*	
H10C	0.5491	0.1665	0.5899	0.062*	
N1	0.33898 (16)	0.45419 (19)	0.65168 (14)	0.0243 (5)	
N2	0.16111 (17)	0.4091 (2)	0.66472 (16)	0.0316 (5)	
H2A	0.1473	0.4850	0.6889	0.038*	
H2B	0.1110	0.3531	0.6558	0.038*	
O1	0.43180 (14)	0.65465 (18)	0.70442 (15)	0.0359 (5)	
H1	0.3833	0.6015	0.6985	0.054*	

### Atomic displacement parameters (Å^2^)

**Table:**

	*U*^11^	*U*^22^	*U*^33^	*U*^12^	*U*^13^	*U*^23^
S1	0.0270 (4)	0.0238 (4)	0.0301 (4)	−0.0019 (2)	−0.0005 (2)	−0.0051 (2)
C1	0.0263 (12)	0.0229 (12)	0.0234 (11)	−0.0001 (9)	−0.0006 (9)	−0.0005 (9)
C2	0.0234 (12)	0.0238 (12)	0.0225 (11)	0.0026 (9)	0.0002 (9)	0.0014 (9)
C3	0.0258 (12)	0.0266 (13)	0.0274 (12)	0.0006 (10)	0.0004 (9)	−0.0009 (9)
C4	0.0236 (12)	0.0264 (12)	0.0223 (11)	0.0008 (9)	−0.0014 (9)	0.0036 (9)
C5	0.0243 (12)	0.0270 (12)	0.0277 (12)	0.0022 (10)	−0.0017 (9)	0.0022 (10)
C6	0.0313 (14)	0.0312 (13)	0.0321 (13)	−0.0040 (11)	−0.0043 (11)	0.0010 (10)
C7	0.0285 (14)	0.0421 (16)	0.0328 (13)	−0.0106 (11)	−0.0026 (10)	0.0045 (12)
C8	0.0250 (14)	0.0473 (17)	0.0385 (14)	−0.0006 (12)	0.0061 (11)	0.0019 (13)
C9	0.0274 (13)	0.0335 (14)	0.0333 (13)	0.0010 (11)	0.0047 (10)	−0.0017 (11)
C10	0.0338 (15)	0.0321 (15)	0.0581 (18)	0.0044 (12)	0.0047 (13)	−0.0131 (13)
N1	0.0221 (10)	0.0226 (10)	0.0282 (10)	0.0003 (8)	0.0016 (8)	−0.0015 (8)
N2	0.0228 (11)	0.0298 (11)	0.0422 (12)	−0.0017 (9)	0.0024 (9)	−0.0075 (10)
O1	0.0242 (9)	0.0275 (10)	0.0561 (12)	0.0006 (7)	−0.0007 (8)	−0.0113 (9)

### Geometric parameters (Å, °)

**Table:**

S1—C1	1.730 (2)	C6—H6	0.9500
S1—C3	1.742 (3)	C7—C8	1.390 (4)
C1—N1	1.302 (3)	C7—H7	0.9500
C1—N2	1.359 (3)	C8—C9	1.382 (4)
C2—C3	1.364 (4)	C8—H8	0.9500
C2—N1	1.403 (3)	C9—H9	0.9500
C2—C4	1.477 (3)	C10—H10A	0.9800
C3—C10	1.498 (4)	C10—H10B	0.9800
C4—C9	1.405 (3)	C10—H10C	0.9800
C4—C5	1.414 (4)	N2—H2A	0.8800
C5—O1	1.368 (3)	N2—H2B	0.8800
C5—C6	1.397 (4)	O1—H1	0.8400
C6—C7	1.376 (4)		
			
C1—S1—C3	90.41 (12)	C6—C7—H7	120.1
N1—C1—N2	124.6 (2)	C8—C7—H7	120.1
N1—C1—S1	113.39 (19)	C7—C8—C9	119.6 (3)
N2—C1—S1	121.98 (19)	C7—C8—H8	120.2
C3—C2—N1	114.3 (2)	C9—C8—H8	120.2
C3—C2—C4	129.6 (2)	C8—C9—C4	122.4 (3)
N1—C2—C4	116.1 (2)	C8—C9—H9	118.8
C2—C3—C10	133.6 (2)	C4—C9—H9	118.8
C2—C3—S1	109.35 (19)	C3—C10—H10A	109.5
C10—C3—S1	117.0 (2)	C3—C10—H10B	109.5
C9—C4—C5	116.7 (2)	H10A—C10—H10B	109.5
C9—C4—C2	122.1 (2)	C3—C10—H10C	109.5
C5—C4—C2	121.2 (2)	H10A—C10—H10C	109.5
O1—C5—C6	116.4 (2)	H10B—C10—H10C	109.5
O1—C5—C4	122.8 (2)	C1—N1—C2	112.6 (2)
C6—C5—C4	120.8 (2)	C1—N2—H2A	120.0
C7—C6—C5	120.6 (3)	C1—N2—H2B	120.0
C7—C6—H6	119.7	H2A—N2—H2B	120.0
C5—C6—H6	119.7	C5—O1—H1	109.5
C6—C7—C8	119.9 (2)		
			
C3—S1—C1—N1	0.48 (19)	C9—C4—C5—C6	−1.2 (3)
C3—S1—C1—N2	177.9 (2)	C2—C4—C5—C6	179.3 (2)
N1—C2—C3—C10	−175.7 (3)	O1—C5—C6—C7	−179.8 (2)
C4—C2—C3—C10	3.6 (5)	C4—C5—C6—C7	0.3 (4)
N1—C2—C3—S1	1.0 (3)	C5—C6—C7—C8	0.5 (4)
C4—C2—C3—S1	−179.6 (2)	C6—C7—C8—C9	−0.2 (4)
C1—S1—C3—C2	−0.85 (19)	C7—C8—C9—C4	−0.8 (4)
C1—S1—C3—C10	176.5 (2)	C5—C4—C9—C8	1.5 (4)
C3—C2—C4—C9	3.0 (4)	C2—C4—C9—C8	−179.0 (2)
N1—C2—C4—C9	−177.7 (2)	N2—C1—N1—C2	−177.3 (2)
C3—C2—C4—C5	−177.6 (2)	S1—C1—N1—C2	0.0 (3)
N1—C2—C4—C5	1.8 (3)	C3—C2—N1—C1	−0.7 (3)
C9—C4—C5—O1	178.9 (2)	C4—C2—N1—C1	179.9 (2)
C2—C4—C5—O1	−0.6 (4)		

### Hydrogen-bond geometry (Å, °)

**Table:**

*D*—H···*A*	*D*—H	H···*A*	*D*···*A*	*D*—H···*A*
O1—H1···N1	0.84	1.77	2.521 (3)	148
N2—H2B···O1^i^	0.88	2.25	2.961 (3)	138

Symmetry codes: (i) −*x*+1/2, *y*−1/2, *z*.
